# Radiomics-integrated machine learning framework for quantitative breast cancer diagnosis

**DOI:** 10.3389/frai.2026.1752413

**Published:** 2026-03-19

**Authors:** Kaavya Jayakrishnan, Nitish Katal

**Affiliations:** 1 School of Electronics Engineering, Vellore Institute of Technology, Chennai, Tamil Nadu, India; 2 Center for Cyber Physical Systems, Vellore Institute of Technology, Chennai, Tamil Nadu, India

**Keywords:** breast cancer, convolutional neural networks, machine learning, radiomics, support vector machine, UNet

## Abstract

**Background and objective:**

Breast cancer is one of the most common cancer types affecting women worldwide, and its early detection is crucial for effective treatment. The proposed study offers an automated pipeline that uses deep learning, radiomics, and machine learning to segment and classify breast tumors. The pipeline takes ultrasound scans as input, segments the tumor using UNet, and uses the predicted segment to compute the radiomic features, which are then given as input to the machine learning models for classification.

**Methods:**

In the first phase of the proposed study, the ultrasound scans and the ground truth masks in the BUSI dataset, obtained by the radiologist, are used to extract the radiomic features, followed by training the machine learning (ML) models. These results are used as benchmarks to evaluate the performance and efficacy of the proposed segmentation model. The use of radiomics bridges the gap between medical imaging and quantitative analysis. The features extracted by the proposed system are shape-based and therefore provide morphological information about tumor cells. In the second phase, an automated deep learning pipeline is proposed, in which the UNet is trained to effectively segment tumorous regions in ultrasound scans. Once the segmentation mask is obtained, it is used to compute radiomic features, and the ML algorithms are trained to classify the tumor as benign or malignant.

**Results:**

The proposed UNet-Radiomics-ML framework achieves performance when compared to the benchmark results obtained in the first phase. The proposed framework achieves a mean IoU of 0.94231 and a classification accuracy of 97.8% for the testing data.

**Conclusions:**

The results obtained from this study truly have the potential to positively impact the diagnosis of breast cancer. The use of automated models like UNet for segmentation, radiomics for feature extraction, and ML algorithms for classification will help reduce human intervention and narrow the diagnostic process, yielding quick results.

## 1 Introduction

Breast cancer is one of the most common cancer types affecting women worldwide and is the 2^nd^ leading cause of death amongst women in the USA (American Cancer Society, 2018). Early detection and accurate diagnosis are crucial for improving treatment planning and increasing patients' survival rates. To enable early diagnosis of breast cancer, several computer-based methods are proposed in the literature (Zebari et al., 2021). Currently, as deep learning is dominant in medical imaging, it has been widely reported in the literature for breast cancer diagnosis and classification (Afrin et al., 2023; Nasser and Yusof, 2023; Luo et al., 2024). The performance of the deep learning model depends on the image type, size, and characteristics (Yusoff et al., 2023). Many approaches, such as mammography, X-Rays, Computed Tomography, and ultrasound, are being used as medical imaging modalities for diagnosis; some studies have focused on the introduction of reference-free methods to analyze RNA-seq data for cancer diagnosis (Eralp and Sefer, 2024). Although mammography can be used for early cancer detection, ultrasound scanning is often preferred as an imaging modality because it is noninvasive. The conventional method uses ultrasound scans to manually check tissue abnormalities and relies on manual interpretation, which can be unreliable in the early stages of cancer (Luo et al., 2024). Magnetic resonance imaging (MRI) provides better pathophysiological information about tissue characteristics but is not cost-effective compared to mammography Adam et al. (2023). So, MRI analysis is reserved for the cases that require further clarification after initial screening using mammography and ultrasound.

The use of artificial intelligence (AI) has shown promising results in medical diagnosis, whether for early disease detection or for segmenting cancerous lesions. Thus, the integration of AI into medical imaging can improve the accuracy and reliability of early diagnosis. These AI algorithms can analyze and learn quickly from vast amounts of data and are adaptable; thus, they can aid healthcare professionals with valuable insights that might have been missed during manual inspection and interpretation. The integration of deep learning for breast cancer diagnosis is well studied in the literature (Afrin et al., 2023; Nasser and Yusof, 2023; Luo et al., 2024; Adam et al., 2023), and currently offered solutions can be divided into two main categories: a) classification of the tumors from the medical images and b) the segmentation of the tumor lesion. The classification models primarily employ convolutional neural networks to determine the nature of the tumor and categorize it as benign or malignant (Shen et al., 2019). Whereas segmentation models, such as UNet, aim to accurately localize and measure lesion size (Rezaei, 2021). These deep learning models have shown promising results in improving diagnostic efficacy, reducing dependence on manual inspection, and potentially leading to early detection (Abhisheka et al., 2023).

The field of radiomics further enhances medical imaging by extracting and analyzing quantitative features of tumor characteristics, such as shape, texture, and intensity, from medical images (Pesapane et al., 2023). Integrating radiomics with deep learning will aid in better understanding and diagnosis, and facilitate faster and more efficient medical image processing, as parameters such as the type of breast cancer, its subtype, and grade can be easily identified using radiomics, thereby accelerating diagnosis (Panico et al., 2023; Oh et al., 2023). The radiomic features can be computed using the DICOM images, where the 3D scan as well as the volumetric segmentation obtained of the lesion by the radiologist, which provides quantitative details for the tumor's shape, texture, and intensity, and can be used to train the machine learning models for classification of the lesion (Corredor et al., 2023; Mahmood et al., 2024).

Deep radiomics integrates deep learning with radiomics to enable deep learning algorithms to extract, analyze, and interpret the complex correlations among quantitative features obtained from medical images. This integration enhances the accuracy, reliability, interpretability, and efficiency of medical image analysis for diagnosis. One of the key features of deep radiomics is its ability to analyze vast amounts of medical imaging data, and the integration of deep learning will facilitate hierarchical learning, where these deep learning algorithms will be able to identify and correlate the underlying key patterns and features, to offer more comprehensive and precise assessments of the disease characteristics. Furthermore, by automating the workflow with deep learning, the extraction and analysis will reduce reliance on manual interpretation. Deep radiomics is being used widely across medical specialties, such as oncology, neurology, and cardiology; specifically in oncology, deep radiomics can aid in tumor characterization and subtype classification (Scapicchio et al., 2021; Avanzo et al., 2020).

In this study, a complete automated framework for breast cancer diagnosis is presented, which uses deep learning, radiomics, and machine learning to segment and classify breast tumors. The study proposes an automated framework that takes scans as input, segments the tumor using UNet, and uses the predicted segment to compute radiomic features, which are then used as input to machine learning models for classification. In the first phase of the proposed study, the ultrasound scans and ground-truth segmentation masks obtained by the radiologist in the BUSI dataset are used to extract radiomic features, which are then used to train machine learning (ML) models. These results are used as benchmarks to evaluate the performance and efficacy of the proposed framework. In the second phase, an automated DL framework is proposed, in which UNet is trained to effectively segment tumorous regions in the scans. Once the predicted masks are obtained, they are used to compute the radiomic features, which are then given as input to ML algorithms to classify the tumor as benign or malignant. The classification results are compared not only with the ML models trained in phase 1 but also with conventional CNN models, such as GoogleNet, ResNet50, and InceptionResNetV2. It has been found that the proposed UNet-Radiomics-ML framework achieves comparable performance to the benchmark results obtained in phase 1. It has been found that, in phase 1, the support vector machine (SVM) achieved the best classification accuracy of 91.7% and the UNet model used for segmentation obtained a validation accuracy of 94.83% and a Mean IoU value of 94.231. Our novelty lies in a sequential two-phase framework that automates the radiomics workflow. Unlike pure DL approaches or pure radiomics approaches (which rely on tedious manual annotation), our pipeline uses UNet to generate robust Regions of Interest (ROI), which are then fed into a Radiomics-ML classifier to capture key tissue heterogeneity. This hybrid design combines the automation of deep learning with the interpretability of traditional machine learning.

The main contributions of the study are as follows:

- The research proposes an integrated approach that combines deep learning (specifically UNet for segmentation), radiomics for quantitative analysis of tumor characteristics from medical images, and machine learning algorithms for classification. Allowing for a more comprehensive analysis of breast tumors by accurate segmentation, quantitative feature extraction, and classification based on extracted features.
- The proposed framework takes ultrasound scans as input, followed by segmentation using the UNet to effectively segment the tumor regions. The predicted masks are then used to compute radiomic features, and the ML algorithms have been trained to classify tumors as benign or malignant.
- The classification efficacy of the proposed model has been evaluated with conventional CNN models like GoogleNet, ResNet50, and InceptionResNetV2; as well as with the ML algorithms with radiomics features as input. The obtained results demonstrate that the proposed UNet-Radiomics-ML framework achieves comparable performance to the benchmark results.

The manuscript is divided into the following sections: Section 1 presents the introduction and problem definition, followed by the literature review in Section 2. Section 3 details the proposed solution, discussing the framework's modules and data pre-processing methods, as well as various deep learning architectures. Section 4 presents the qualitative and quantitative analysis of the obtained results, followed by a comparison of the proposed method with existing state-of-the-art methods in Section 5, and concludes the proposed study.

## 2 Literature review

Over the past decade, the integration of AI has advanced and shown promising results in medical diagnosis, playing an important role in disease diagnosis across various medical specialties, such as oncology, neurology, and cardiology, thereby improving the accuracy and reliability of early diagnosis. Specifically in oncology, the AI is aiding in the localization, characterization, and classification of the tumors; and broadly spans around two main categories: a) the classification of the tumors from the medical images using ML algorithms and CNNs; and b) the segmentation of the tumor lesion using UNet, FCNs, mask RCNNs, k-means clustering, etc.

The application of deep learning to breast cancer diagnosis has been well studied in the literature (Afrin et al., 2023; Nasser and Yusof, 2023; Luo et al., 2024; Yusoff et al., 2023; Adam et al., 2023). In Zhu et al. (2021), the use of CNNs, namely TNet and BNet, is presented for thyroid and breast lesion classification, respectively. The models have been trained on a custom dataset collected by the authors in a hospital, comprising 672 breast and 719 thyroid scans. TNet and BNet exhibited an average accuracy of 86.5% and 89%, respectively. Three radiologists were used to validate further the models' performance, which demonstrated the model's ability for precise lesion classification. In Deka et al. (2026) proposed a hybrid feature selection and classification framework for breast cancer using mammography images, wherein a median filter is used for image enhancement, followed by adaptive thresholding for segmentation and CatBoost for classification.

In Moon et al. (2020); Karthik et al. (2022), hybrid feature extraction modules were proposed; wherein in Moon et al. (2020) three parallel branches comprising AlexNet, ResNet50, and MobileNetV2 were considered, followed by mRMR for feature selection and SVM and kNN for classification, whereas in Karthik et al. (2022) a combination of four branches featuring AlexNet, ResNet, ShuffleNet, and MobileNet was considered to propose five hybrid models for classification. In Ting et al. (2019), a 30-layer CNN is proposed featuring feature-wise data augmentation and an SSD-based detector for lesion localization. In Karthik et al. (2022), the authors proposed a Gaussian dropout-based stacked ensemble CNN to classify breast ultrasound scans into different tumor types, comprising three stacked subnetworks. In Sirjani et al. (2023), an InceptionV3-inspired architecture is proposed that introduces residual inception modules.

SMM-UNet (Li et al., 2025) features a selective fusion and a multi-scale fusion mamba module in a UNet-inspired architecture for breast tumor segmentation. FET-UNet features parallel ResNet34 and Swin Transformer feature extractors in the encoder of the UNet-inspired architecture. EMGA-Net (Huang et al., 2025) multi-scale group-mix (MGM) attention and edge feature enhancement (EFE) modules to feature an attention-driven encoder-decoder architecture. In Zhang et al. (2025), the encoder section comprises multi-scale convolutional blocks with attention modules, and the decoder section includes edge refinement layers for breast tumor segmentation.

In Chen et al. (2023), RRCNet is proposed for segmentation, with an architecture comprising three modules: SegNet, a supervision module, and two residual modules for missed and false detections. In He et al. (2023); Eroğlu et al. (2021), a hybrid CNN-transformer network, HCTNet, is proposed, featuring a UNet-inspired architecture with transformer encoder blocks and spatial cross-attention, and the decoder features residual connections. In Podda et al. (2022), a deep learning pipeline is proposed, featuring a combination of VGG19, ResNet50, DenseNet121 with UNet for segmentation and CNN models like InceptionV3, Xception, and Densenet201 for classification.

Table 1 provides a summary of the existing methods for the classification and segmentation of breast cancer.

**Table 1:** Comparison of the existing literature.

**References**	**Method**	**Dataset**	**Accuracy**	**Specificity**	**Sensitivity**	**Other metrics**	**Focus**
Zhu et al. (2021)	CNN (TNet, BNet)	Thyroid, Breast	86.5% (TNet) 89% (BNet)	–	86.6% (TNet breast)	–	Classification
Moon et al. (2020)	CNN feature extraction + SVM/kNN	BUSI	95.6% (SVM)	–	–	–	Classification
He et al. (2023)	Ensemble CNN	SNUH, BUSI	91.10% (SNUH) 90.77% (BUSI)	95.77% (SNUH)	85.14% (SNUH)	0.9697 AUC (SNUH) 0.9489 AUC (BUSI)	Classification
Ting et al. (2019)	CNNI-BCC (30-layer CNN)	MIAS	90.50%	90.71%	89.47%	–	Classification
Sahu et al. (2023)	Hybrid CNN (AlexNet, ResNet, ShuffleNet, MobileNet)	mini-DDSM, BUSI, BUS2	94.23% (Hybrid 5)	96.15% (Hybrid 5)	92.31% (Hybrid 5)	96% Precision (Hybrid 5)	Classification
Karthik et al. (2022)	Stacked Ensemble CNN with Gaussian Dropout	Combined	87.64%	–	–	87.68% F1-score	Classification
Sirjani et al. (2023)	Modified InceptionV3	BUSI, BUS, Thailand (Public), Iran (Private)	81%	–	71% Recall	0.18 RMSE	Classification
Eroğlu et al. (2021)	HCTNet	BUS, BUSI, BUS 2017 Dataset B	96.94%	–	–	82% Dice 82.14% Recall	Segmentation
Chen et al. (2023)	RRCNet (Refinement Residual ConvNet)	BUSI, BUSI Dataset B	–	–	–	**Dice** 78.85% (B) 70.79% (M) **Precision** 78.73% (B) 69.72% (M) **Jaccard** 71.83% (B) 59.95% (M)	Segmentation
Podda et al. (2022)	CNN (VGG19, ResNet50, DenseNet121) + UNet, InceptionV3, Xception, DenseNet201 (Classif.)	BUSI (development), OASBUD (validation)	91.14% Accuracy (Classif.)	–	–	82.60% Dice (Seg.)	Segmentation & Classification

## 3 Proposed solution

### 3.1 Overview of automated deep learning framework

**Figure 1:**
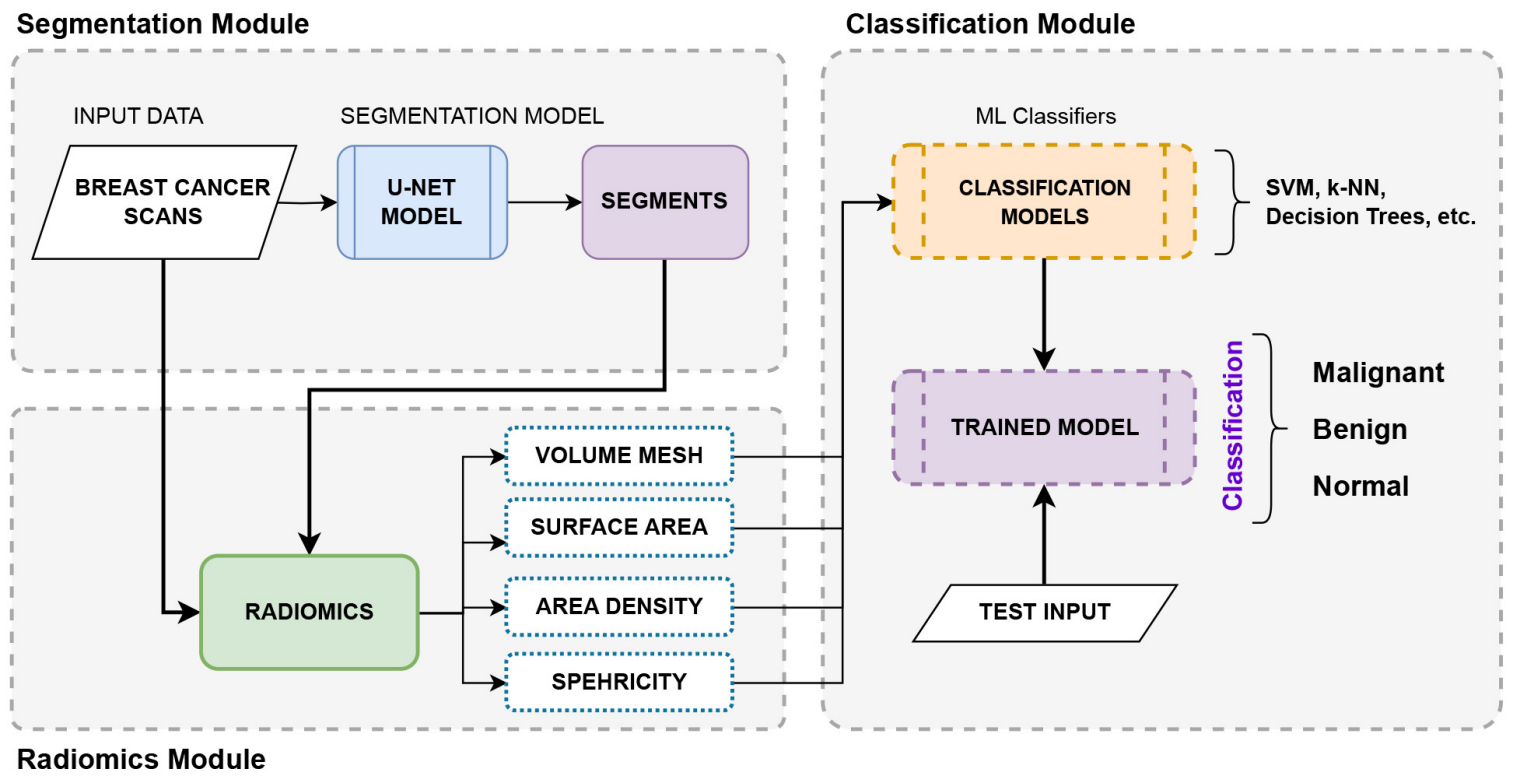
Overview of the proposed automated deep learning framework.

The proposed system comprises three major modules, based on deep learning and traditional machine learning classifiers, that function as a fully automated framework for segmenting and classifying tumors. The proposed study employs a novel radiomics approach that extracts features from medical images to improve classification accuracy. The proposed integrated approach to breast cancer segmentation and classification is developed to address the challenges posed by manual disease diagnosis and analysis. The automated deep learning framework follows a logical step-by-step implementation beginning with a segmentation module, a radiomic feature extraction module, and finally a classification module, as shown in Figure 1.

Module-1 performs semantic segmentation using the UNet architecture to identify and segment tumor regions. The model is trained on ultrasound scans and ground-truth masks obtained by radiologists to accurately compare and segment the images. This task is crucial as it lays the foundation for the upcoming modules in the proposed framework. These segmented binary masks will serve as input to module 2, where radiomic features will be extracted.

In Module 2, the segments obtained in Module 1 are used to extract the radiomic features using the ultrasound images and the predicted masks. Radiomics is the quantitative analysis of features extracted from medical scans non-invasively. The proposed model considers only shape-based radiomic features, such as surface area, volume mesh, sphericity, compactness, area density, etc., to study these scans morphologically and leverage these features to classify them into different types of breast cancers. Furthermore, in clinical practice, the shape of breast tumors in a medical image is very informative for the purpose of classifying breast tumors as benign or malignant. Because shape is a strong predictor for this specific clinical task (benign tumors tend to be round/oval, while malignant ones are irregular). Thus, radiologists often classify breast lesions based on specific 2D planes (the transverse and longitudinal views) that capture the maximum diameter or the most suspicious morphology. The use of 2D features in the proposed study aligns with this standard diagnostic workflow, wherein it focuses on the morphological patterns present in the representative slices.

The segmentation results and radiomic features obtained from Module 1 and Module 2, respectively, are combined to form an integrated representation of the ultrasound image dataset of breast tumors. This will serve as input for Module 3, where the final classification occurs. In Module 3, the extracted radiomic features are used to train the machine learning algorithms to classify the lesions as benign or malignant. In this module, classical ML algorithms, such as SVMs, k-NN, neural networks, and decision trees, have been used for classification. Furthermore, to establish the accuracy of the proposed method, a comparison has been presented, comparing the obtained results with classification results from pre-trained convolutional neural network (CNN) models such as GoogleNet, ResNet50, and InceptionResNetV2 to classify tumors using only the original ultrasound scan dataset.

### 3.2 Segmentation strategy

**Figure 2:**
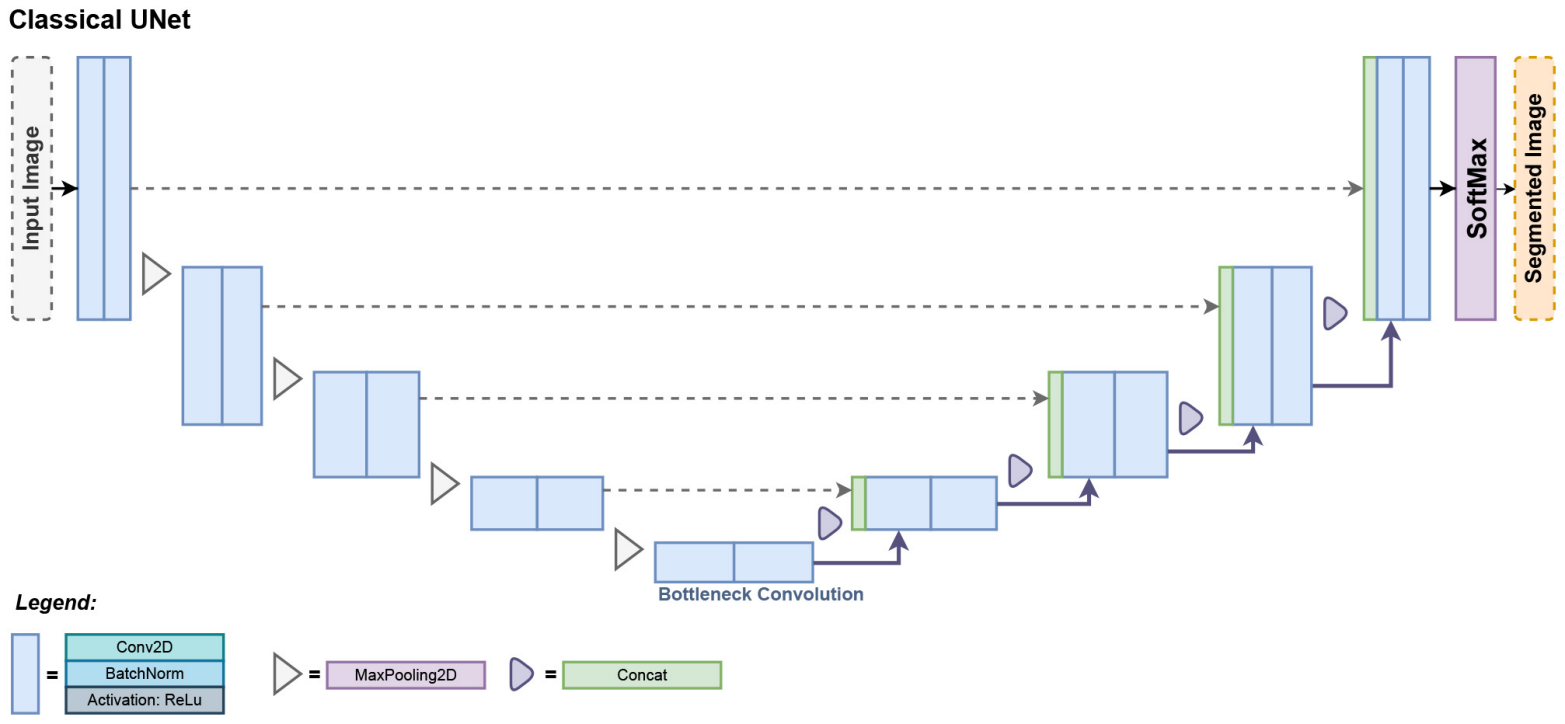
Standard UNet architecture.

The proposed automated deep learning framework uses UNet to accurately segment lesions and abnormalities in breast ultrasound scans. UNet architecture features a distinctive encoder-decoder structure as depicted in Figure 2. Ronneberger et al. (2015) first introduced a UNet architecture, specifically designed for biomedical image segmentation, which features convolutional and pooling layers and a few skip connections between two major paths, i.e., the encoder (contracting path) and decoder (expansive path).

The encoder path features a sequence of convolutional layers followed by max-pooling layers to down-sample the input's spatial dimensions, while gradually increasing the number of feature channels to extract as many features as possible. It is used to capture contextual and semantic information from the input images. Whereas the decoder path aids in recovering the spatial dimensions of feature maps and generating the final segmentation results. This path plays a crucial role, as it accurately outlines the tumor's boundaries across the entire scan. At each level of the decoder path, feature map concatenation occurs with corresponding feature maps from the encoder via skip connections. These skip connections enable integration of advanced semantic features and low-level spatial information. By incorporating these connections, the UNet model overcomes the challenges faced by traditional CNN models, namely maintaining spatial information across model layers and accurately segmenting objects with well-defined boundaries.

**Figure 3:**
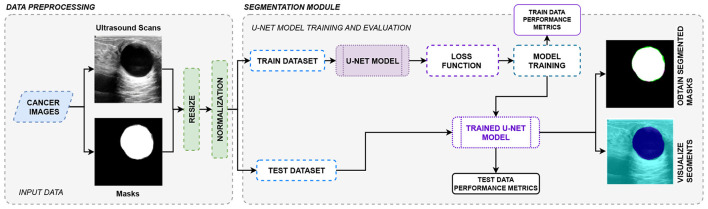
Proposed segmentation module using UNet architecture.

Figure 3 shows the proposed segmentation module using UNet, which is trained on the BUSI dataset (Al-Dhabyani et al., 2020) comprising ultrasound scans of breast cancer-affected breasts. These images are accompanied by their corresponding ground-truth segmentation masks, which serve as references during the training of the segmentation model. These masks are created and validated by expert radiologists who manually annotate regions of interest (ROIs) in the images, carefully defining the boundaries of tumors and other abnormalities in the breast tissue.

To begin the training process, the entire dataset is divided into a training set and a validation set. The training set comprises most of the data. It is used to optimize the parameters of the UNet model by iteratively minimizing errors at each subsequent layer via backpropagation. Data augmentation techniques such as rotation, flipping, resizing, and normalization scaling are applied to the training data to improve the generalization capabilities of the trained model. During each iteration, the input images are compared with their corresponding ground-truth masks, and any encountered errors are computed and corrected in the next iteration via iterative backpropagation. A suitable loss function, such as Dice Loss, is chosen to measure the deviation between the original masks and the predicted segments.

The calculated error in each iteration is propagated backward through the network, and the model's parameters are revised to minimize the error in subsequent iterations. After each iteration during training, the validation data are used to evaluate the model's performance. After completing the training process, the UNet model can perform semantic segmentation on unseen breast ultrasound scans. So, given any breast cancer scan that the model has not been trained on, the model will process the image through the different layers of the architecture to generate a binary segmentation mask of the input.

### 3.3 Radiomic feature extraction

Radiomics is a field within medical image analysis that involves extracting a large number of significant features from 3D medical images to quantify the geometric and morphological characteristics of tumor cells in diagnostic procedures. This non-invasive approach was first introduced by Aerts et al. (2014) to demonstrate its potential in predicting clinical outcomes from medical imaging data. Radiomics can be used to extract three types of medical features from 3D images: lesion shape, intensity, and texture.

**Figure 4:**
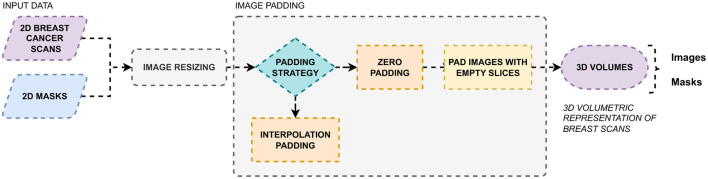
Preprocessing stage for conversion of 2D ultrasound images to 3D volumes.

In the proposed automated deep learning framework, Module 2, as shown in Figure 1, focuses on radiomic feature extraction to extract shape features for breast cancer diagnosis from ultrasound images. Radiomics is traditionally focused on 3D images such as Computed Tomography (CT) and Magnetic Resonance Imaging (MRI) scans. However, the BUSI dataset used in the present study consists of 2D ultrasound scans, and for radiomic feature extraction, data preprocessing and conversion are mandatory to transform 2D planes to 3D volumes. The process is illustrated in Figure 4, which starts with the collection of breast ultrasound scans and their corresponding segments, obtained either from radiologists, as in Phase 1 of the study, or segmented by the trained UNet model. The resolution and dimensions of the input data are updated, and a suitable padding strategy is selected for converting to 3D volumes. There are two commonly followed padding techniques, zero padding and interpolation padding. For this proposed system, zero padding is used, adding empty slices above and below each 2D image to create 3D volumes. These empty slices are typically pixels with constant values, such as zeros.

**Figure 5:**
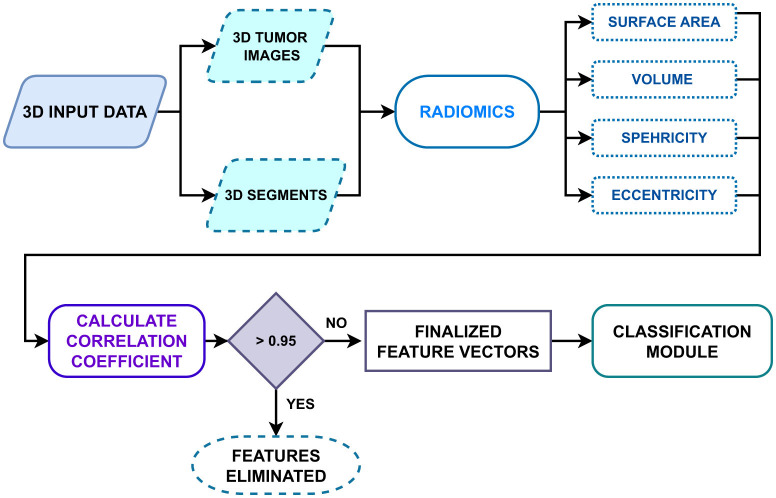
Radiomic feature extraction framework for classification task.

After obtaining the 3D representations of the breast cancer images, the next step in the workflow is to extract shape-based features from them by comparing the segmented 3D masks, as shown in Figure 5. Volume, surface area, compactness, surface-to-volume ratio, spherical disproportion, sphericity, elongation, flatness, eccentricity, and convexity are a few commonly extracted shape-based features. It is essential to evaluate the relevance and redundancy of these extracted features before finalizing the features to consider for the ML-based classification module of the proposed framework. Some features may exhibit high correlation; hence, a common approach to feature selection is to compute correlation coefficients between pairs of features. If the calculated value between two features exceeds a pre-set threshold, one of the features in each pair will be removed from the feature set. The predefined threshold in this case is set to 0.95; any feature with a correlation greater than 0.95 is considered redundant and removed. This optimized feature set will enable improved results for the forthcoming classification models in Module 3 of the proposed automated deep learning framework. The generalized radiomic feature extraction workflow is discussed in Table 2.

**Table 2:** Radiomics feature extraction workflow.

**Phase**	**Procedure**	**Key methods**	**Output**
Image acquisition	Standardized image collection	CT/MRI/PET with documented acquisition parameters	DICOM images
Preprocessing	Image standardization	Resampling, intensity normalization, denoising	Standardized images
Segmentation	ROI delineation	Manual, semi-automatic, or AI-based segmentation	ROI mask
Discretization	Gray-level binning	Fixed bin width or fixed bin number	Quantized image
Feature extraction	Quantitative feature computation	Shape, first-order, GLCM, GLRLM, GLSZM, NGTDM, GLDM, wavelet (IBSI-compliant software)	Feature matrix
Feature selection	Dimensionality reduction	Correlation filtering, mRMR, LASSO, PCA	Selected features
Model dev. & Validation	Predictive modeling and evaluation	Machine learning classifiers; cross-validation; ROC/AUC analysis	Performance metrics

### 3.4 Deep learning and machine learning models for classification

The features extracted using radiomics in Module 2 play a crucial role in the classification of breast tumors into malignant and benign. The proposed system explores the application of machine learning algorithms to classify cancer types. The study uses traditional algorithms such as SVMs, KNNs, neural networks, and decision trees.

The primary step in the machine learning framework is selecting the features to train the model; in the current study, the required radiomic features have already been obtained and finalized from the previous modules of the proposed framework. The selected data is then split into two or more sub-datasets: a training set, a validation set, and a small portion held out as a testing set. The training dataset enables the model to learn important patterns and leverage supervised learning by identifying deviations between the provided features and target outcomes. Hyperparameter tuning is necessary and performed by evaluating the training process on a validation dataset. A portion of the dataset is kept unseen during model training and is introduced specifically for optimization. The test dataset is used to evaluate the final trained model's performance on various metrics such as accuracy, recall, precision, sensitivity, F1 score, and specificity.

In the present study, three ML algorithms, KNNs, SVMs, and Decision Trees, have been used for classification. K-Nearest Neighbors (KNNs) is a supervised machine learning algorithm that operates based on a ‘k” value, which is determined by the dataset and the similarity between classes. For breast cancer classification, the training data are used to compute the features that determine ‘k”. The KNN algorithm computes distances between each radiomic feature vector and groups similar vectors together. The process is iterative, with each iteration introducing a new tumor case to the model. Upon training in each iteration, every time a new case is introduced, a probability is calculated, where if 7 out of 10 of the previous cases are closer to the ‘k' value and are labeled as benign, then the new case, if closer to the ‘k' value, will be automatically classified as benign. The performance of the KNN model depends heavily on the linearity of the input data, the distance metric, the value of 'k', and the quality of the input data.

Support Vector Machines (SVMs) are supervised machine learning classifiers that can be trained to classify breast tumor lesions, based on radiomic features, into benign, malignant, and normal classes. It operates on a hyperplane that maximizes the distance between classes. SVMs perform well on high-dimensional medical data and are therefore particularly useful when using radiomic features extracted from 3D volumes of breast lesions. Decision Trees (DT) is a tree-based classification algorithm that recursively partitions feature vectors into smaller subsets based on the input features. A hierarchical decision-making model can be developed using the obtained features to classify breast tumors.

**Figure 6:**
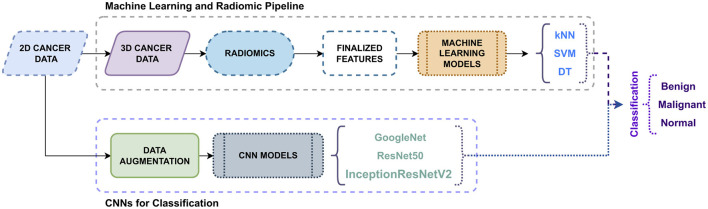
Proposed classification approach.

In addition to the traditional machine learning classifiers, the proposed framework conducts a comparative study using deep learning models, such as Convolutional Neural Networks (CNNs), on the original 2D images obtained from the dataset, as shown in Figure 6. CNNs are advanced classification models that are well-suited for medical image classification. For breast tumor classification, the chosen CNN models are GoogleNet, ResNet50, and InceptionResNetV2, which will be trained directly on the original data rather than on the extracted radiomic features and UNet-derived segments. CNNs can learn features directly from the input data without having to extract them separately. The performance of these CNN models that produce classification outputs without feature extraction is compared with that of traditional classifiers applied to radiomic features.

**Figure 7:**
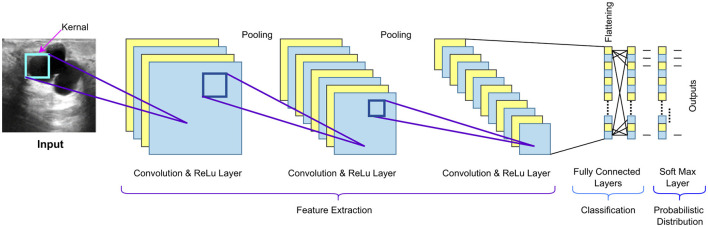
Typical CNN architecture for classification.

Figure 7 depicts the typical architecture of a convolutional neural network (CNN) for the classification of biomedical images. The workflow begins with the input image being passed through multiple convolution layers, where each layer performs automated feature extraction by applying filters of appropriate size. The output of the convolution layers is a feature map that has captured different parameters and characteristics essential for classification from the input image. A non-linear activation function, such as the Rectified Linear Unit (ReLU), is applied to the feature maps to learn more complex features. The feature maps are downsampled using pooling layers to retain important information while reducing their spatial dimensions. The final feature maps are converted into a single vector, which is passed through one or more fully connected layers, where the extracted features from all the layers are combined. The softmax layer is used to compute the probabilities of each class (benign, malignant, normal) given the features, after which the final classification occurs.

**Figure 8:**
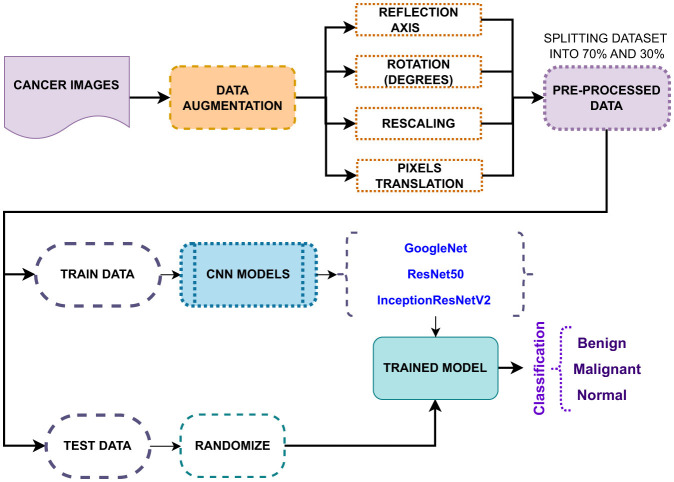
Proposed CNN classification models.

For the comparative study, a common CNN classification workflow is followed, as shown in Figure 8. GoogleNet is a CNN model with 144 layers, known as InceptionV1. Its key approach is the usage of inception modules, which allow efficient computation and improved performance. These inception modules comprise multiple convolution filters of sizes corresponding to the input data. GoogleNet has two auxiliary layers connected to the intermediate layers to mitigate vanishing gradients during training. ResNet50 is another CNN model used in this comparative study. It has a total of 177 layers, and the key aspect of this model is its residual blocks, which address the problem faced by deep neural networks, such as high training errors and vanishing gradients. The residual blocks have skip connections, which automatically add a few inputs from the layer to the output layer. This model has a bottleneck architecture in each residual block. ResNet50, due to its deep architecture, has the potential to precisely classify breast tumor images and achieve a more generalized model than shallower networks. InceptionResNetV2 is a hybrid CNN model that combines the inception blocks of GoogleNet and residual blocks of ResNet50 to form an 824-layer deep neural network. These combined blocks comprise multiple parallel convolutional paths with different filter sizes. Due to its size, the model's computational complexity is very demanding. To reduce this complexity, factorized convolution layers are introduced, resulting in fewer parameters for the model to remember. Although the depth of the model allows for capturing intricate patterns and enabling enhanced classification, it has the disadvantage of over-fitting.

## 4 Results and discussions

### 4.1 BUSI dataset

The dataset used is the Breast Ultrasound Images (BUSI) (Al-Dhabyani et al., 2020). This dataset was collected in 2018 by performing ultrasound imaging on women affected by breast cancer between the ages of 25 and 75 years old. A total of 600 patients were considered for the study, and the dataset consists of 780 scans. The average image dimensions are 500 × 500 and are in the Portable Network Graphics (PNG) format. Each ultrasound scan includes its corresponding ground-truth masks in the dataset. The dataset has been categorized into 3 classes, namely Benign, Malignant, and Normal. There are 437 scans of benign tumors, 210 scans of malignant tumors, and 133 scans of normal breast scans. The BUSI dataset has been widely used in the literature (Zhu et al., 2021; Moon et al., 2020; Sahu et al., 2023; Karthik et al., 2022; Sirjani et al., 2023; Eroğlu et al., 2021; Chen et al., 2023; Podda et al., 2022; Li et al., 2025; Huang et al., 2025; Zhang et al., 2025).

### 4.2 Framework used, software and hardware details

The development and testing of the proposed framework have been done on the 2023b release of MATLAB. This study primarily uses the Image Processing, Deep Learning, Machine Learning, Classification Learner, and Radiomics toolboxes for various tasks during development. The development has been carried out on a Windows 11 workstation, with an i9 processor, Nvidia 3060 12 GB GPU, and 32 GB of RAM.

### 4.3 Performance metrics

The efficacy of the proposed automated deep learning framework is evaluated using multiple performance metrics for both segmentation and classification tasks. The metrics calculated are Global Accuracy, Mean Accuracy, Mean Intersection Over Union (IoU), Weighted IoU, Mean BF-Score, Sensitivity, Specificity, and Area Under the Receiver Operating Characteristic Curve (AUC-ROC).

- *Global accuracy*: Global accuracy measures the overall correctness of the model's predictions across all classes. High global accuracy indicates the model's efficiency in distinguishing between cancerous and non-cancerous cells. The general mathematical formula for Global Accuracy is:
$$Global\;Accuracy=\frac{TP+TN}{TP+TN+FP+FN}$$
  Where TP represents true positive, TN represents true negative, FP represents false positive, and FN represents false negative.
- *Mean accuracy*: Mean accuracy is calculated by taking the average of all the accuracy values obtained throughout the training process for each class in the input dataset, where each class is given equal weights. A high mean accuracy indicates the model's efficiency in distinguishing between cancerous and non-cancerous cells. The general mathematical formula for Mean Accuracy is:
$$Mean\;Accuracy=\frac{1}{C}*\sum \left(\frac{TP_{C}}{TP_{C}+TN_{C}+FP_{C}+FN_{C}}\right)$$
- *Mean intersection over union (Mean IoU)*: Mean IoU measures the overlap that occurs between the ground truth segments and the predicted segments, averaged over all classes present in the dataset. It is a metric used to evaluate how accurately the segmentation model predicts by overlaying its predictions on the ground-truth masks. The general mathematical formula for Mean IoU is:
$$Mean\;IOU=\frac{1}{C}*\sum \left(\frac{TP_{C}}{TP_{C}+FP_{C}+FN_{C}}\right)$$
- *Weighted intersection over union (weighted IoU)*: Weighted IoU is calculated by going through each class, and based on the frequency of the classes, weights are allotted. Then, an average of all the IoU values is calculated. The general mathematical formula for Weighted IoU is:
$$Weighted\;IOU=\frac{1}{N}*\sum \left(N_{C}*\frac{TP_{C}}{TP_{C}+FP_{C}+FN_{C}}\right)$$
- *Mean BF-score*: It is a metric that combines precision and recall into a single score with the same weights given to both. A high Mean BF-Score indicated that the model can maintain a good balance between identifying tumors correctly and avoiding false positives. The general mathematical formula for Mean BF-Score is:
$$Mean\;BF\;Score=\frac{2\;*Mean\;Precision\;*Mean\;Recall}{Mean\;Precision\;+Mean\;Recall}$$
  where $Mean\;Precision=\frac{1}{C}*\sum \frac{TP_{C}}{TP_{C}+FP_{C}}$ and $Mean\;Recall=\frac{1}{C}*\sum \frac{TP_{C}}{TP_{C}+FN_{C}}$
- *Sensitivity and specificity*: Sensitivity is known as recall and is a measure of the proportion of actual positives correctly identified by the model, while Specificity is a measure of the proportion of actual negatives identified by the model. The general mathematical formula for Sensitivity and Specificity is as follows:
$$Sensitivity=\frac{TP}{TP+FN},\;Specificity=\frac{TN}{TN+FP}$$
- *Area under the receiver operating characteristic curve (AUC-ROC)*: AUC-ROC is calculated by plotting the true positive rate (TPR) or sensitivity against false positive rate (FPR) at various classification thresholds. It is a measure of evaluating the model's ability to distinguish between positive and negative classes on all classification thresholds. A high AUC-ROC value indicates the model's ability to classify between different classes efficiently and can rank it based on different thresholds.

### 4.4 Analysis of the obtained results

#### 4.4.1 *Phase 1*: Radiomics + ML ground truth

**Figure 9:**
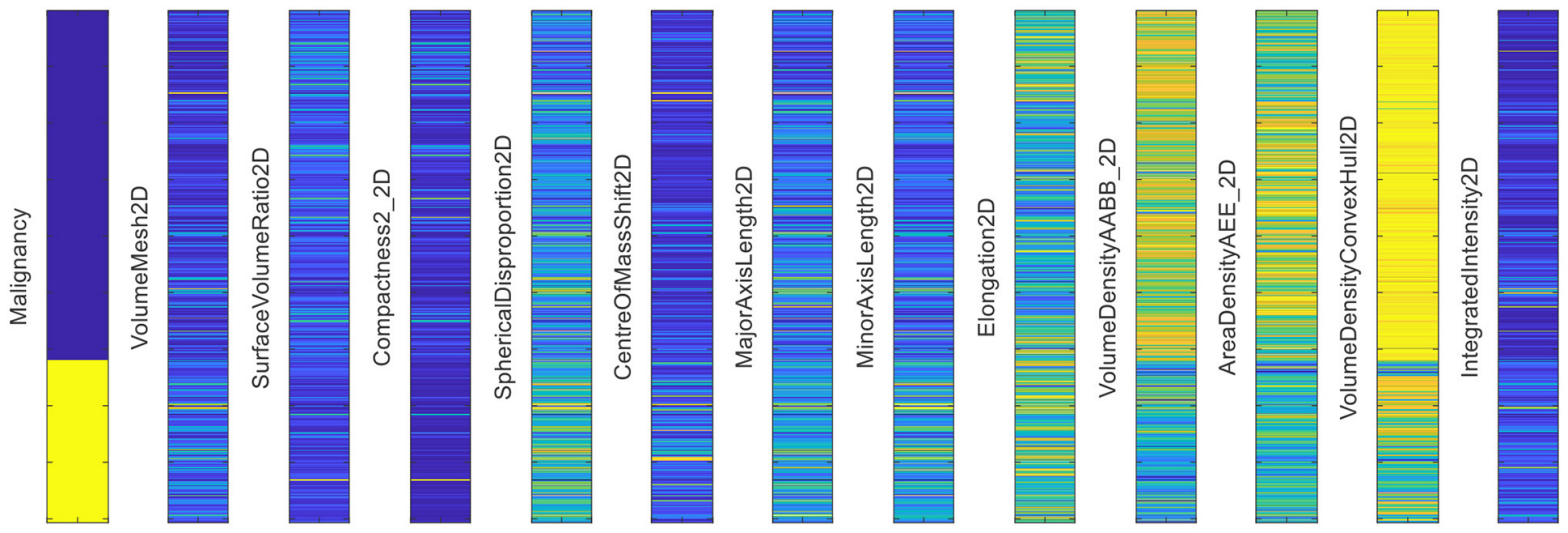
Radiomic features extracted from the BUSI dataset.

**Figure 10:**
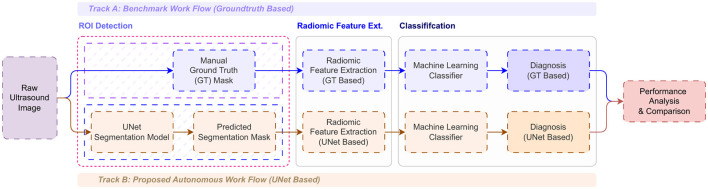
Comparison between the two approaches considered in the proposed work.

In the phase 1, as discussed in the Section 3.2, the radiomic features are extracted for the BUSI dataset, where breast ultrasound scans and its corresponding segments obtained either from the radiologists, are used for extracting the shape-based radiomic features as shown in Figure 5, followed by feature selection where the highly correlated features were discarded by calculating the correlation coefficients between pairs of features. If the calculated value between two features exceeds a pre-set threshold of 0.95, one of the features in each pair will be removed from the feature set. The obtained features are visualized in the Figure 9. A clear demarcation between the two approaches is highlighted in Figure 10.

The obtained radiomic features are then used to train machine learning algorithms using the Classification Learner application in MATLAB, with a stratified 15% samples for 5-fold validation and 10% of the data reserved for testing. Stratification was enabled to preserve class proportions across training, validation, and test sets, and the same data split was consistently used across all ML models to ensure fair comparison. Given a total of 647 samples (437 benign, 210 malignant), the held-out test set contained approximately 97 samples (66 benign and 31 malignant), while the remaining 550 samples were used for 5-fold cross-validation.

**Figure 11:**
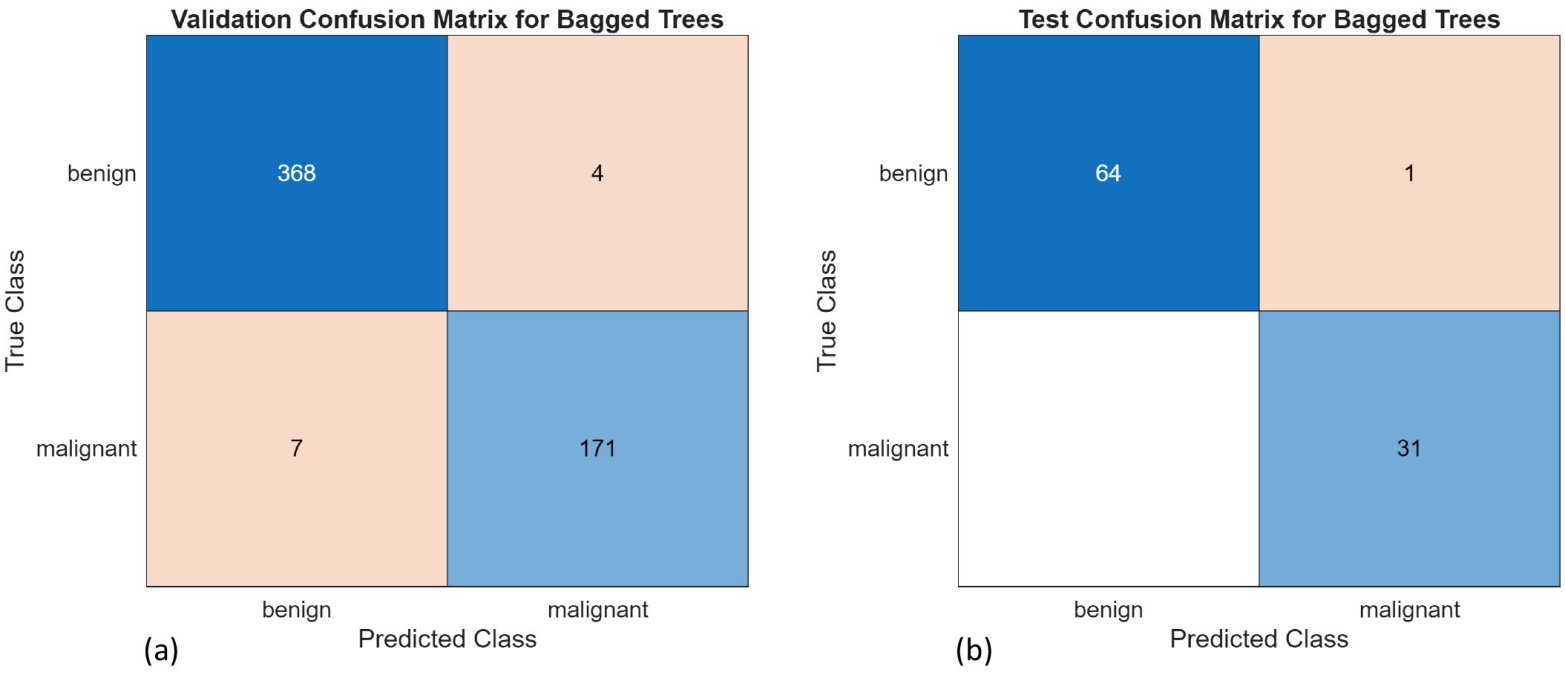
Confusion matrices for the bagged trees for **(a)** validation and **(b)** test set.

**Figure 12:**
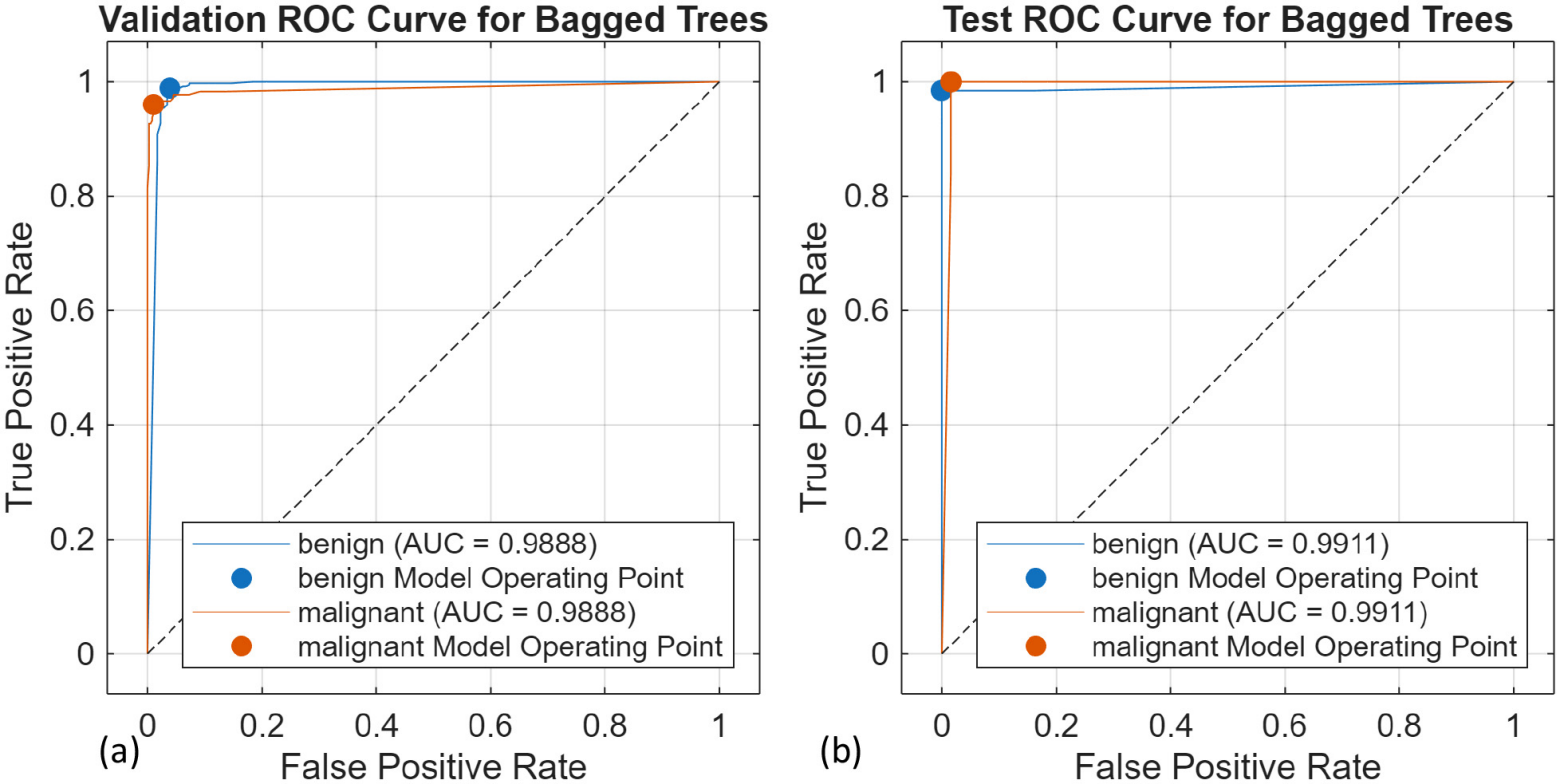
ROC-AUC curve for the bagged trees for **(a)** validation and **(b)** test set.

A total of 10 different algorithms were trained; among them, SVM, k-NN, and decision trees achieved the best validation and test accuracy. The bagged trees achieved the highest validation and test accuracies of 98% and 98.96%, respectively. The obtained results are presented in Table 3. Figures 11a, b are the confusion matrix results of validation and testing sets, respectively. Figures 12a, b represent the ROC Curve for validation and testing sets of Method-1, respectively.

**Table 3:** Performance of the machine learning algorithms for the breast cancer classification on the BUSI dataset considering the radiomic features.

**S. No**.	**Model**	**Validation accuracy**	**Testing accuracy**
1	Radiomics + ensemble (bagged trees)	98	98.96
2	Radiomics + SVM (quadratic SVM)	97.64	98.96
3	Radiomics + neural network	96	98.96
4	Radiomics + trees (coarse)	97.46	97.92
5	Radiomics + KNN (fine)	94.55	96.88

#### 4.4.2 *Phase 2*: UNet + radiomics + ML models

In Phase 2 of the study, an automated framework, as discussed in Section 3.1, has been proposed. The framework consists of three modules: (a) Segmentation Module, (b) Radiomics Extraction Module, and (c) Classification Module, as shown in Figure 1. Module 1 is responsible for semantic segmentation to localize and segment tumor regions in ultrasound images, using the UNet architecture. The UNet model is trained on ground-truth masks obtained by radiologists to accurately compare and segment the images. These segmented binary masks will serve as input to Module 2, where radiomic features will be extracted. In Module 2, the segments obtained in Module 1 are used to extract the radiomic features from the medical images. The segmentation results and radiomic features obtained from Module 1 and Module 2, respectively, are combined to form an integrated representation of the ultrasound image dataset of breast tumors. This will serve as input for Module 3, where the final classification occurs. In Module 3, the extracted radiomic features are used to train the machine learning algorithms to classify the lesions as benign, malignant, or normal. In this module, classical ML algorithms, such as SVMs, k-NN, and decision trees, have been used for classification. Furthermore, to establish the accuracy of the proposed method, a comparison has been presented, comparing the obtained results with classification results from pre-trained convolutional neural network (CNN) models such as GoogleNet, ResNet50, and InceptionResNetV2 to classify tumors using only the original ultrasound scan dataset.

##### 4.4.2.1 UNet segmentation results

The segmentation module uses a UNet architecture (Figure 2) to segment tumorous lesions (Figure 3).

**Figure 13:**
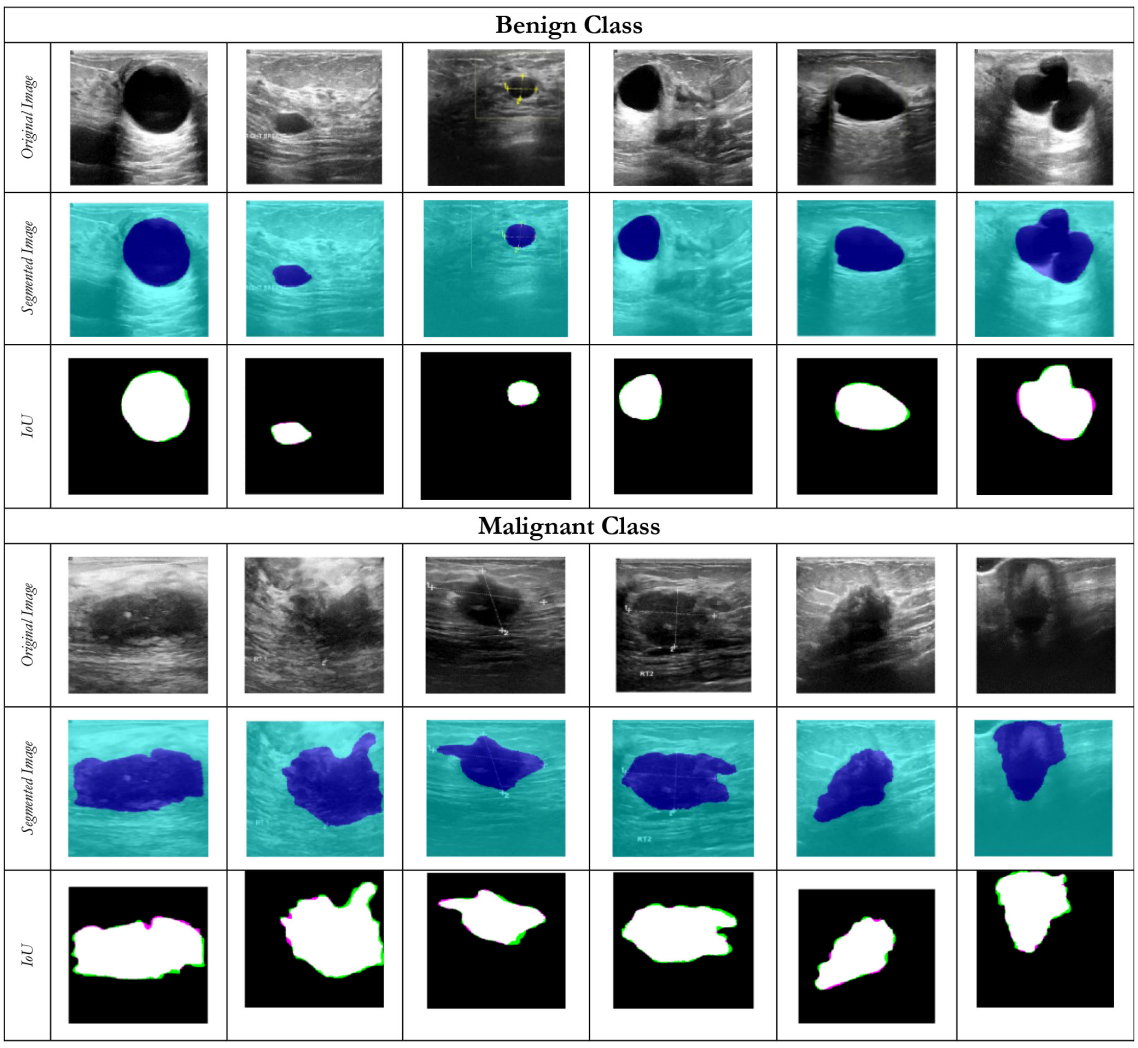
A comparison between the original tumor image and its segments for both classes in the BUSI dataset.

**Figure 14:**
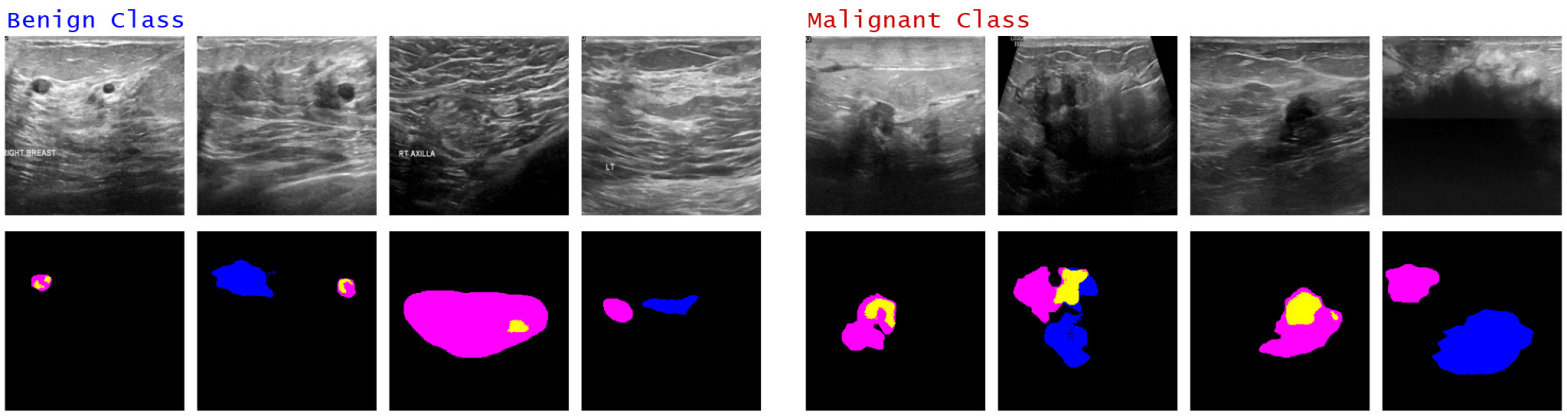
Some of the failure cases for both the classes.

For segmentation, the dataset is split into training and validation sets, with 92.5% of the data used for training and 7.5% for validation. In the proposed research, a larger training subset (92.5%) is intentionally adopted; our core hypothesis is that precise tumor segmentation is the essential prerequisite for the downstream success of the ML models. A segmentation fidelity will ensure that subsequent radiomics extraction and machine learning models receive high-quality, anatomically accurate regions of interest, thereby improving the reliability of the final results. Thus, by allocating a larger percentage to the training set, we ensured the segmentation network could capture sufficient morphological variance to generalize effectively. Table 4 represents the training progress of the proposed UNet segmentation Model for breast tumor segmentation, showing the model's training and validation accuracy across epochs and iterations. The accuracy increases as training progresses, indicating the model's efficiency in learning to segment tumors accurately. After a few epochs, it stabilizes, showing that the model has learned the required features to segment the images. The number of epochs chosen is 170. Figure 13 shows the segments for each class. Figure 14 shows some of the segmentation failure cases of the model for each class.

**Table 4:** Training progress of UNet segmentation model.

**Epoch**	**Iteration**	**Training accuracy**	**Validation accuracy**
1	1	0.880	0.125
50	900	0.953	0.935
70	1260	0.969	0.949
100	1790	0.986	0.953
130	2340	0.993	0.948
170	3060	0.995	0.948

Table 5 represents the performance metrics of semantic segmentation done by the UNet model on the BUSI dataset, which was split into training and prediction sets. The metrics include Global Accuracy, Mean Accuracy, Mean IoU, Weighted IoU, and Mean BF-Score. The high values of these metrics indicate the segmentation model's efficiency in accurately producing segments on any unseen tumor image.

**Table 5:** Semantic segmentation performance metrics of UNet.

**Type**	**Global accuracy**	**Mean accuracy**	**Mean IoU**	**Weighted IoU**	**Mean BF-Score**
Training	0.99368	0.96787	0.96161	0.98745	0.92875
Prediction	0.99288	0.95154	0.94231	0.98590	0.93717

##### 4.4.2.2 UNet + radiomics + machine learning

**Figure 15:**
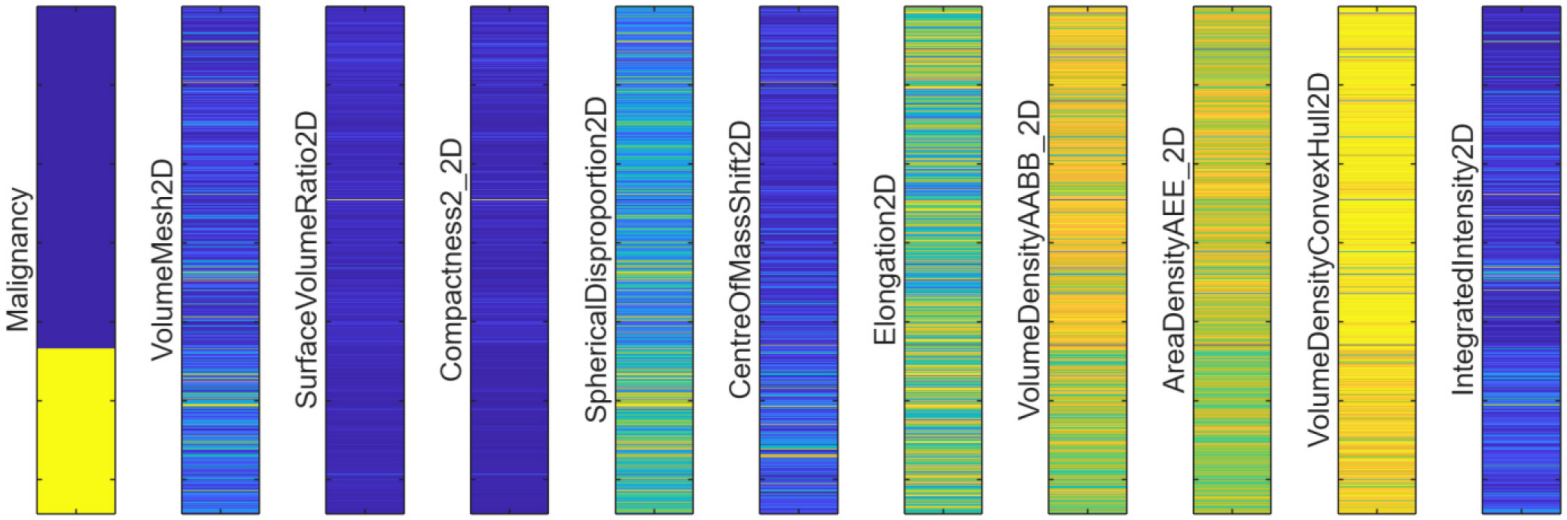
Radiomic features extracted from the BUSI dataset, using the UNet masks.

**Figure 16:**
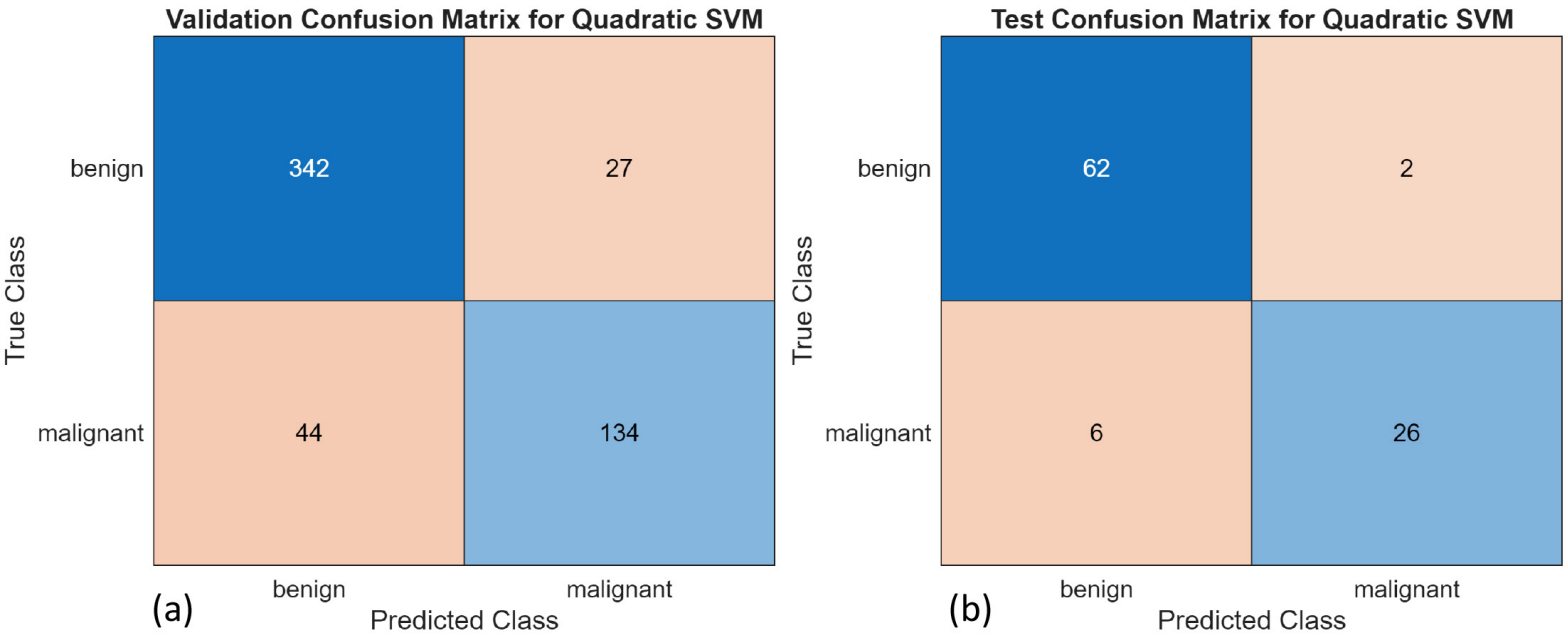
Confusion matrices for the Quadratic SVM for **(a)** validation and **(b)** test dataset.

**Figure 17:**
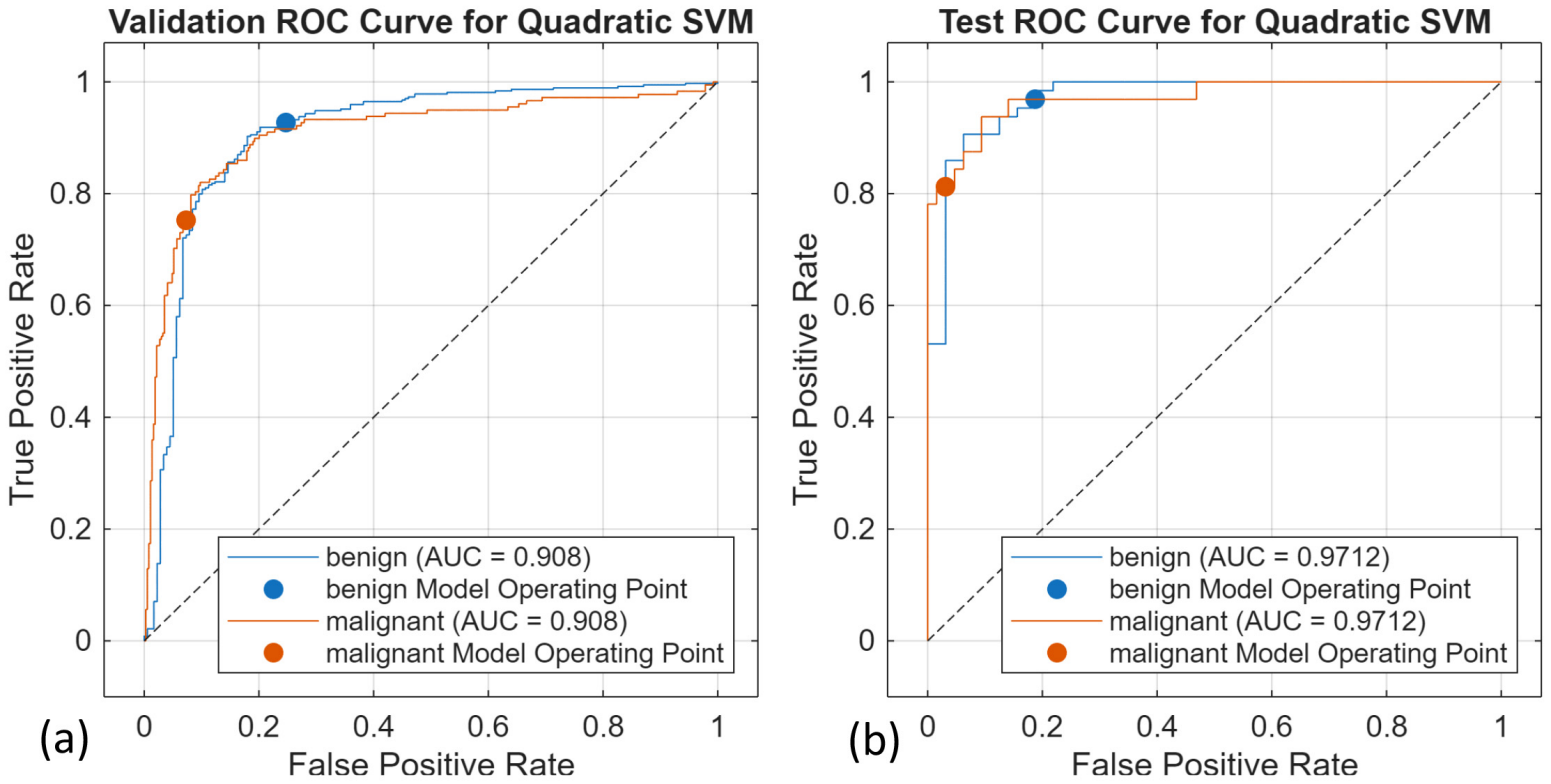
ROC-AUC curve for the Quadratic SVM for **(a)** validation and **(b)** test set.

The same procedure as discussed in 4.4.1 is applied to the segments obtained from UNet, and these segments are then used to compute the radiomic features. The selected radiomic features computed using the UNet segments are shown in Figure 15. These features were again used to train 10 classical machine learning algorithms, and out of which three models, kNN, SVM, and decision trees, gave the best performance. Table 6 shows that the quadratic SVM yields a validation accuracy of 87.02% and a testing accuracy of 91.67%. Bagged trees achieve equally good validation accuracy of 86.29% and testing accuracy of 94.79%. The slight difference in performance between the two radiomic feature approaches is attributed to the manual segmentation performed by expert radiologists and the automated segmentation performed by deep learning models. Although manual segmentation is more accurate, it is often time-consuming; thus, automated segmentation models speed up the process and yield reliable segments. The SVM models consistently outperformed all the other models, regardless of the feature extraction approach. This is due to the model's ability to handle high-dimensional features and distinguish between decision boundaries of different classes even when they are non-linearly separable. Figures 16a, b are the confusion matrix results of validation and testing sets, respectively. Figures 17a, b represent the ROC Curve for validation and testing sets of Method 1, respectively.

**Table 6:** Performance of the machine learning algorithms for the breast cancer classification on the BUSI dataset considering the radiomic features extracted using the UNet segments.

**S. No**.	**Model**	**Validation accuracy**	**Testing accuracy**
1	UNet segments + radiomics + SVM (quadratic)	87.02	91.67
2	UNet segments + radiomics + Ensemble (bagged trees)	86.29	94.79
3	UNet segments + Radiomics + neural network	83.36	91.67
4	UNet segments + Radiomics + tree (Fine)	83.91	90.63
5	UNet segments + Radiomics + fine KNN	83.73	90.63

#### 4.4.3 CNN model results

**Figure 18:**
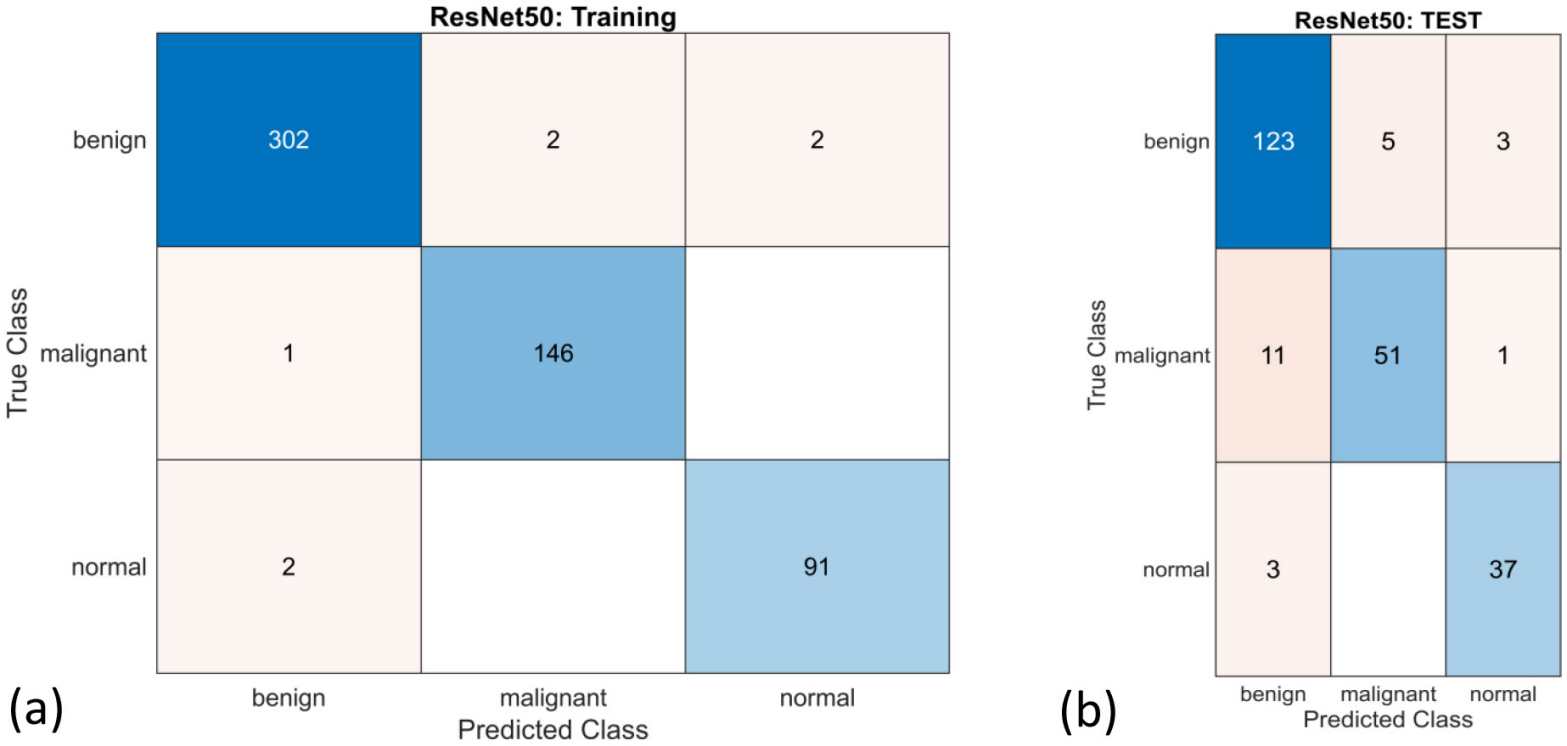
Confusion matrices for the ResNet50 model for **(a)** training and **(b)** test dataset.

**Figure 19:**
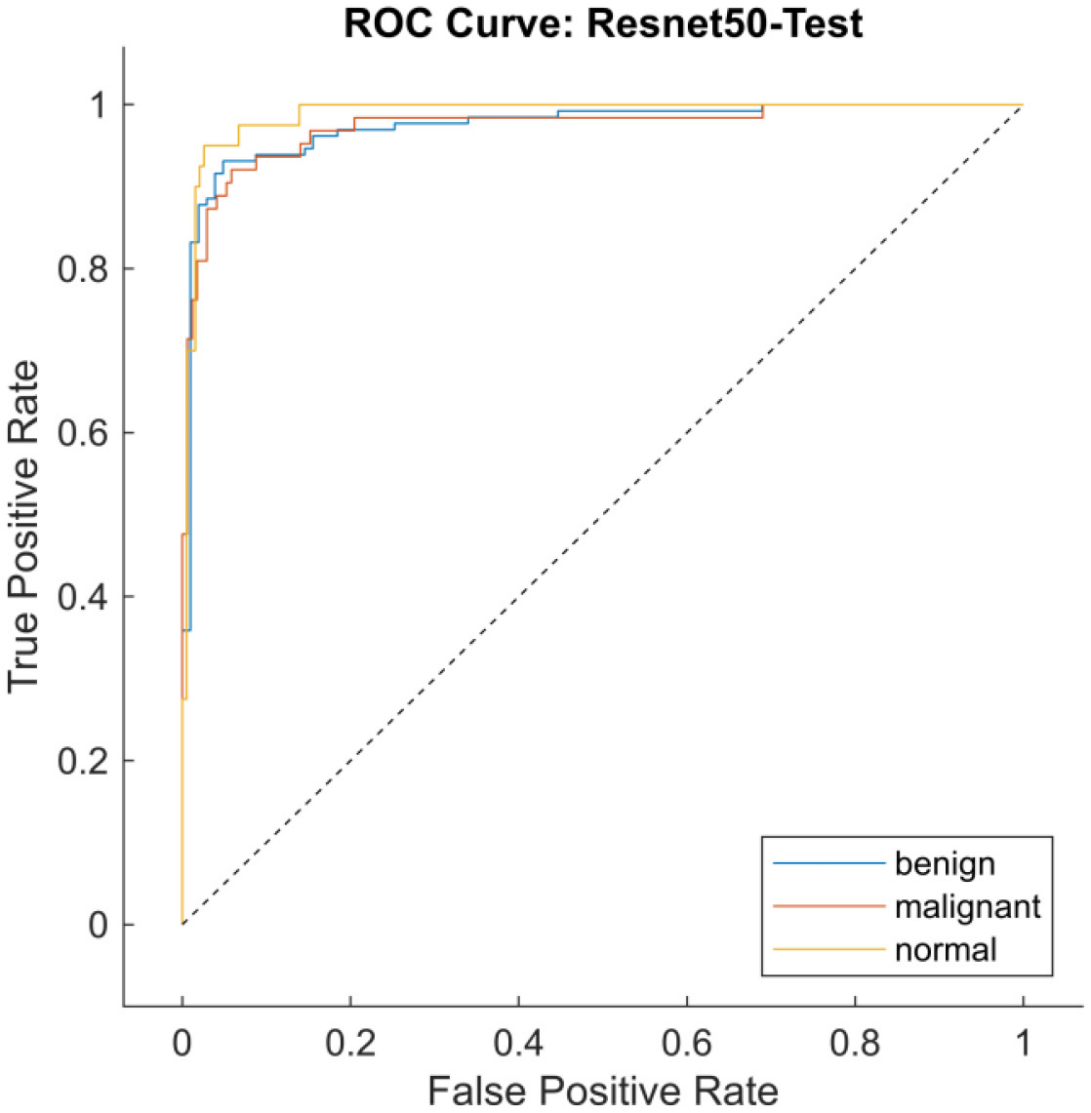
ROC-AUC curve for the ResNet50 for training and test dataset.

Table 7 represents the performance metrics of three different CNN models used for the classification of breast cancer on the original BUSI dataset. The CNN models evaluated are GoogleNet, ResNet50, and InceptionResNetV2, with InceptionResNetV2 achieving the highest training accuracy of 99.4%, indicating superior performance on the training data. However, ResNet50 achieved the highest validation accuracy of 90.1%, indicating that the model is more generalizable and reliable across datasets for classification. Figures 18a, b are the confusion matrix results of training and testing sets, respectively, for the ResNet50 model. Figure 19 represents the ROC Curve for all three classes for the ResNet50 model.

**Table 7:** Performance of the CNN models for the breast cancer classification on the BUSI dataset.

**Model**	**Training accuracy**	**Validation accuracy**	**Sensitivity**	**Specificity**
GoogleNet	0.932	0.867	0.940	0.967
ResNet50	0.987	0.901	0.986	0.985
InceptionResNetV2	0.994	0.863	0.995	0.996

## 5 Comparison with existing research

To demonstrate the efficacy of the proposed study, the obtained results are compared with those of existing methods on the BUSI dataset. A few models used hybrid CNNs (Eroğlu et al., 2021), comprising AlexNet, ResNet50, and MobileNetV2, to extract features from the BUSI dataset and perform classification. These features are not used directly; instead, an mRMR feature reduction method is followed, and then SVM and KNN models are used for classification. Computer-aided diagnostic systems (CADs) (Moon et al., 2020) are used to classify tumors as benign or malignant, using VGGNet, ResNet, and DenseNet for classification. It follows an image-fusion approach, which differs from conventional CAD systems. Hybrid CNN (Eroğlu et al., 2021) models used for feature extraction and classification achieved accuracies of 89.5%, 92.1%, and 90.7% for the AlexNet + SVM, ResNet50 + KNN, and MobileNetV2 + SVM models, respectively. CADS [19], used for classification, achieves accuracies of 93.2%, 94.6%, and 95.1% for the VGGNet, ResNet, and DenseNet models.

A fully automated deep learning pipeline for segmentation and classification of breast ultrasound images (Podda et al., 2022) uses UNet for segmentation and CNN models, such as InceptionV3, Xception, and DenseNet201, for classification. It has an accuracy of 94.3% for UNet (Segmentation) + InceptionV3 (Classification), 95.8% for UNet (Segmentation) + Xception (Classification) and 96.2% for UNet (Segmentation) + DenseNet201 (Classification).

The current state-of-the-art models use different CNN architectures to segment and classify the BUSI dataset into malignant, benign, and normal classes. The proposed framework in the current study uses a combination of models, including a deep CNN for segmentation, radiomics for feature extraction, and machine learning for classification, ultimately achieving a test accuracy of 97.8%. Thus, the proposed system's accuracy of 97.8% outperforms the existing methods due to its integrative approach. The proposed system significantly enhances accuracy and efficiency for breast tumor segmentation and classification. The comparison of the proposed framework with the state-of-the-art methods is presented in Table 8. Thus, the accuracy of the proposed system outperforms current state-of-the-art techniques, which are known to rely on a single approach that uses only CNN models for both segmentation and classification.

**Table 8:** Performance comparison of the proposed method with the existing state-of-the-art methods.

**Segmentation**	
**Method**	**Mean IoU**
*Proposed UNet*	0.94231
**Classification**	
**Method**	**Accuracy %**
AlexNet + SVM [25]	89.5
ResNet50 + KNN [25]	92.1
MobileNetV2 + SVM [25]	90.7
VGGNet (CADS) [25]	93.2
ResNet (CADS) [19]	94.6
DenseNet (CADS) [19]	95.1
UNet + InceptionV3 [27]	94.3
UNet + Xception [27]	95.8
UNet + DenseNet201 [27]	96.2
*Proposed System*	Radiomics + SVM: 97.8 (Test) & 97.5 (Val.) UNet + Radiomics + SVM: 97.8 (Test) and 82.1 (Val.)

The proposed automated framework's scalability and generalizability are two important advantages. With separate modules for segmentation, feature extraction, and classification, the proposed architecture is adaptable to different datasets and imaging modalities. The deep CNN segmentation model can be used for a variety of breast imaging data, including magnetic resonance imaging scans, ultrasounds, and mammograms. Moreover, it can be retrained on new datasets. Likewise, the modules for radiomic feature extraction and machine-learning classification can be updated to support different features, enabling the system to operate across diverse clinical conditions.

## 6 Conclusions

The proposed study offers an automated framework that uses deep learning, radiomics, and machine learning to segment and classify breast tumors. This study proposed an automated framework that takes scans as input, segments the tumor using UNet, and uses the predicted segment to compute radiomic features, which are then used as input to machine learning models for classification. The two different classification approaches, the ML algorithms and CNNs, produced good results, but the SVM yielded the best accuracy in both cases of using ground truth masks from the original BUSI dataset and the segments obtained from the proposed UNet model. Since the proposed framework aims to automate segmentation and classification, the validation and testing accuracies obtained with the SVM model outperformed those of all other approaches. Meanwhile, the UNet model used to segment the tumor images performed well at producing automated segments. It achieved a high global accuracy and a high mean IoU, indicating its efficiency in producing accurate segments. Even though the results obtained with the original segments for classification yielded higher accuracies, the proposed system produced promising results and therefore avoids the need for manual segment marking by radiologists. The results obtained from this study truly have the potential to positively impact the diagnosis of breast cancer. The use of automated models like UNet for segmentation, radiomics for feature extraction, and ML algorithms for classification will help reduce human intervention and narrow the diagnostic process, yielding quick results.

## Data Availability

The datasets presented in this study can be found in online repositories. The names of the repository/repositories and accession number(s) can be found below: https://scholar.cu.edu.eg/?q=afahmy/pages/dataset.
